# Economy, migrant labour and sex work: interplay of HIV epidemic drivers in Zimbabwe over three decades

**DOI:** 10.1097/QAD.0000000000002066

**Published:** 2018-12-03

**Authors:** Richard Steen, Jan A.C. Hontelez, Owen Mugurungi, Amon Mpofu, Suzette M. Matthijsse, Sake J. de Vlas, Gina A. Dallabetta, Frances M. Cowan

**Affiliations:** a Department of Public Health, Erasmus MC, University Medical Center Rotterdam, Rotterdam, The Netherlands; b Ministry of Health and Child Care; c National AIDS Council, Harare, Zimbabwe; d BresMed Health Solutions, Utrecht, The Netherlands; e The Bill & Melinda Gates Foundation, Washington DC, USA; f Centre for Sexual Health and HIV AIDS Research (CeSHHAR Zimbabwe), Harare, Zimbabwe; g Liverpool School of Tropical Medicine, Liverpool, United Kingdom.

**Keywords:** AIDS epidemic, economic factors, epidemic drivers, generalized epidemic, HIV, migrant labour, sex work, Zimbabwe

## Abstract

**Background:**

HIV took off rapidly in Zimbabwe during the 1980s. Yet, between 1998 and 2003, as the economy faltered, HIV prevalence declined abruptly and without clear explanation.

**Methods:**

We reviewed epidemiological, behavioural, and economic data over three decades to understand changes in economic conditions, migrant labour and sex work that may account for observed fluctuations in Zimbabwe's HIV epidemic. Potential biases related to changing epidemic paradigms and data sources were examined.

**Results:**

Early studies describe rural poverty, male migrant labour and sex work as conditions facilitating HIV/sexually transmitted infection (STI) transmission. By the mid-1990s, as Zimbabwe's epidemic became more generalized, research focus shifted to general population household surveys. Yet, less than half as many men than women were found at home during surveys in the 1990s, increasing to 80% during the years of economic decline. Other studies suggest that male demand for sex work fell abruptly as migrant workers were laid off, picking up again when the economy rebounded after 2009. Numbers of clients reported by sex workers, and their STI rates, followed similar patterns reaching a nadir in the early 2000s. Studies from 2009 describe a return to more active sex work, linked to increasing client demand, as well as a revitalized programme reaching sex workers.

**Conclusion:**

The importance of the downturn in migrant labour and resultant changes in sex work may be underestimated as drivers of Zimbabwe's rapid HIV incidence and prevalence declines. Household surveys underrepresent populations at the highest risk of HIV/STI acquisition and transmission, and these biases vary with changing economic conditions.

## Abstract 2

Supplemental Digital Content is available in the text

## Introduction

**Fig. 1:**
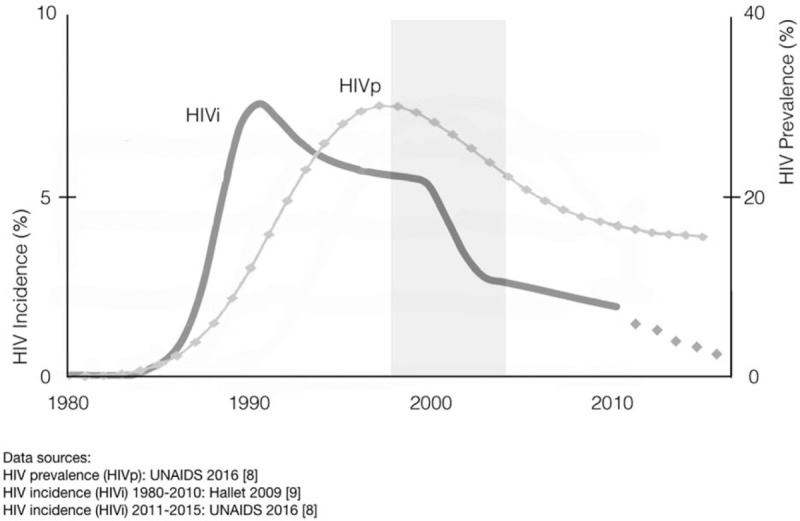
Modelled HIV incidence and prevalence in Zimbabwe [8,9].

Zimbabwe's HIV epidemic emerged in the 1980s and disseminated rapidly across the country [1–3]. HIV incidence peaked in the early 1990s and, by 1995, about one in four Zimbabwean adults was HIV-positive [4]. Incidence slowly declined during the late 1990s, a period when social and economic conditions were starting to regress [5]. Then, between 1998 and 2003, adult HIV incidence and prevalence dropped sharply as the country sank deeper into economic crisis (Fig. 1) [6–9].

Changes in general population sexual behaviours were also reported during this period [9–11]. Yet, concurrent data on higher risk populations and settings are limited, raising important questions about transmission dynamics and causal pathways. If behaviour change contributed to observed epidemiologic trends, what behaviours actually changed, among which people, and how did those changes interrupt transmission enough to impact incidence and prevalence so abruptly?

The importance of sex work and population mobility in early concentrated HIV epidemics, and for other sexually transmitted infections (STIs), is widely recognized (see supplementary appendix). Predominantly male migration (high male-to-female ratio), thought to increase demand for sex work, was associated with larger HIV epidemics. With high rates of partner change and secondary transmission via ‘bridge’ populations, HIV transmission potential in sex work has been estimated to be several hundred times higher than in lower risk networks, even in advanced generalized epidemics.

To the extent that HIV epidemics continue to thrive on interdependent dynamics of sex work, migration and mobility, changes in economic or other conditions that suppress population movement should be reflected in incidence and prevalence trends. In Zimbabwe, the period of rapid HIV prevalence decline coincided with economic shocks that abruptly reduced employment and disrupted migrant labour patterns. Understanding such trends may help clarify epidemic drivers and inform interventions for epidemic control.

## Methods

We reviewed epidemiological, behavioural and economic data to understand historical trends in Zimbabwe's HIV epidemic and underlying population dynamics. References were identified through PubMed for articles published from January 1980 through December 2016, using terms ‘Zimbabwe’ and ‘HIV.’ Programmatic and survey reports were identified through official channels and internet searches. All abstracts were screened and articles mentioning adult HIV/STI transmission, interventions, data or modelling were included. Summary data on economic trends were obtained from the World Bank [5].

## Results

### An early epidemic driven by migrant labour and sex work (circa 1980 to late 1990s)

Phylogenetic and historical evidence (see supplementary appendix) suggest that HIV moved with migrant labour from Léopoldville (Kinshasa) to the Copper Belt of Eastern Congo during the half-century before it first appeared in Zimbabwe. By the time AIDS was recognized, conditions for efficient spread through migrant networks, amplified by long-distance trucking, were well established across the region.

Early studies describe the rapid expansion of HIV across Zimbabwe and initial responses to slow transmission. Conditions seen as facilitating transmission included land reform, rural poverty and male migrant labour, leading to new patterns of sexual relations and multiple partnerships [1,12]. Male demand for sex outside regular partnerships was driven by large-scale migrant labour and met by an ‘almost ubiquitous expectation’ of women to be rewarded for sex outside marriage [2]. Such economic conditions – especially in the transport, mining and commercial farm sectors – led to high rates of infection among economically productive adults. Prevalence among miners was 20–30%, highest at mines along major transport routes [3].

HIV/STI prevention and research during this period focused on sex work. Ethnographic studies described the social and work environment, the importance of improving access to interventions for both sex workers and clients [13,14]. Sex work was seen as playing a critical role in STI transmission generally, and involving sex workers was promoted as good prevention [15]. Most women, on the other hand, were seen to be at risk mainly because of their partners’ behaviours [16]. And those regular male partners appeared to be at greater risk of becoming infected the more time they spent away from home [17].

Limited data suggest that sex work in Zimbabwe in the late 1980s involved high numbers of clients, little condom use and extremely high STI rates. Interviews with disco/bar-based sex workers in Harare revealed that sex workers worked on average 4.4 nights per week and had 2.2 clients per night, whereas clients reported visiting sex workers 7.4 times in the last month. Inconsistent condom use at last paid sex was reported by sex workers (54%) and clients (44%) [13]. In Bulawayo, sex workers reported working 3.6 nights a week, averaged 1.3 clients a night and used condoms with 39% of clients [14].

Zimbabwe demonstrated that peer-based interventions with sex workers, linked to condom and STI services, could improve conditions in sex work, increase condom use and decrease STI incidence [15,18]. These early interventions also sought to increase community participation of sex workers and to reach clients. Zimbabwe was one of the first countries to launch condom social marketing and to adopt STI syndromic case management [18].

Data support a general slowing of sexual transmission in all populations during this period. The number of patients presenting with STI syndromes declined by 60% in Harare between 1992 and 1996, and countrywide by a third between 1989 and 1999 [19]. Following introduction of peer education for sex workers in Mutare in 1991, the number of patients attending health facilities with STIs dropped by one-third within a year [18]. Male condom distribution, free and socially marketed, increased almost three-fold between 1990 and 1999 to 60 million (see supplementary appendix). By the mid-1990s, however, only about one-third of urban-based female sex workers were being reached by interventions [18].

### Programmes and research focus shifts to the general population (circa 1998–2008)

Awareness of rapid HIV dissemination to lower risk populations was growing even as early interventions sought to stem transmission in urban sex work networks. A study conducted among the general population in Harare in 1989 found high rates of premarital, casual and paid sex, while condom use was very low, even among married respondents who reported casual sex [17]. Over a third of married respondents reported living apart from their spouses. An early study from two rural districts reported high rates of STIs, acceptance of men having multiple partners, and of sex work, related to changing socioeconomic conditions [20].

As HIV became entrenched in diverse communities across the country, the attention of both researchers and programmes shifted to the general population. In 2000, WHO defined three epidemic states for surveillance purposes – low, concentrated and generalized – with recommendations for routine data collection (see supplementary appendix) [11,21]. These surveillance classifications, following reported HIV declines in Uganda, influenced programming and policy recommendations across generalized epidemics of Eastern and Southern Africa. In 1999, 2006, 2010 and 2015, the Zimbabwe Demographic and Health Surveys (ZDHS) included modules on HIV, as did the Multiple Indicator Cluster Survey (MICS) in 2014 [22,23]. Yet, surveillance among sub-populations at risk remained spotty, linked to evaluation of specific projects.

**Fig. 2:**
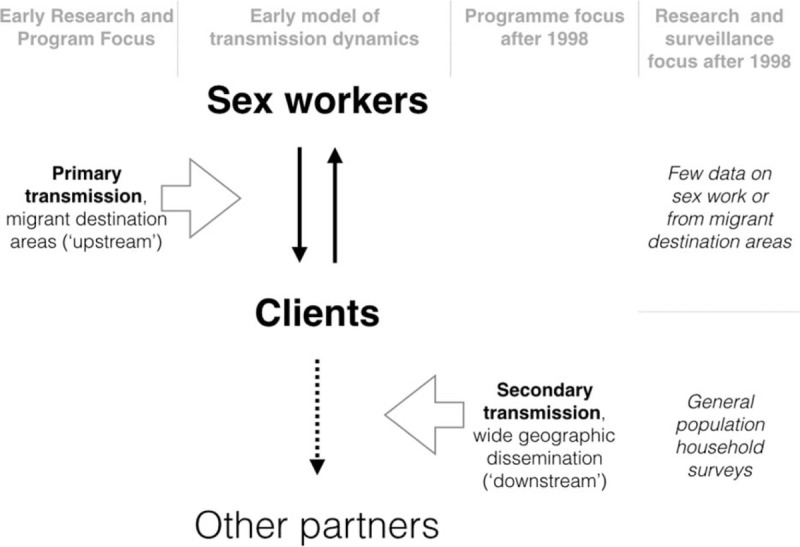
Changing data sources shift attention to the general population.

Nearly half the studies cited in this review were conducted in Manicaland, in an open cohort of four subsistence farming areas, two roadside trading centres, four forestry, tea and coffee estates and two small towns [24]. Populations and conditions in these settings differed greatly from those in urban and other ‘high-transmission’ areas that were the primary focus during the first decade of Zimbabwe's HIV epidemic (Fig. 2).

**Fig. 3:**
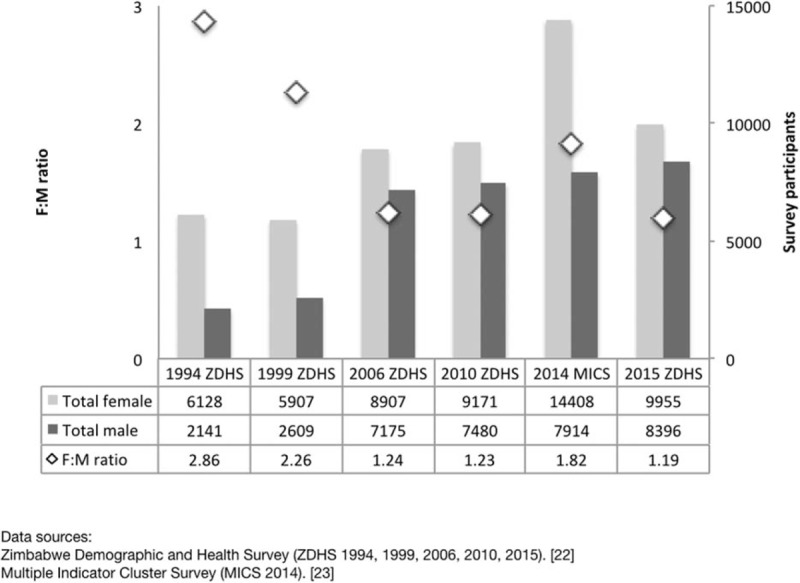
Female-to-male ratio of household survey respondents.

Important biases were also introduced in shifting to household surveys. Men, for example, were underrepresented in ZDHS and MICS, to a large and variable degree (Fig. 3). Less than half as many men (ages 15–54) than women (ages 15–49) were found at home in the 1990s whereas the proportion increased to 80% during the years of economic decline. Where were the missing men, and why do the proportions of men found at home vary so widely over time? What are their risks and how do they influence transmission overall? Could household surveys reflect mainly ‘secondary’ transmission, while missing important epidemic drivers?

Despite such biases, general population data provided copious information about the majority of Zimbabweans and, by the late 1990s, evidence of changing behaviours was appearing [7,25,26]. HIV prevalence was 23% overall in the first round (1998) of the Manicaland cohort [24]. Young women reported male partners who were 5–10 years older. Yet, authors concluded that reducing unprotected sex between men and sex workers, and improving STI services, would lower HIV incidence among young women, and be easier to achieve than reducing unprotected sex between older men and young girls [27].

High-risk sexual behaviour, including sex work, clearly existed in rural cohorts [28,29]. HIV prevalence was higher among women in community centres compared with subsistence farming areas (49.9 versus 24.7%). Prevalence was also higher among migrant agricultural workers compared with other sexually active women (38.8 versus 29.7%) and men (26.4 versus 20.9%). But relating survey findings to sexual networks outside the study area proved more challenging as the latter could only be measured indirectly. Higher prevalence, for example, was found among men who travelled to Harare without their spouses [30]. Another study reported no significant differences in HIV incidence or sexual behaviour between rural-to-urban out-migrants and residents, but was limited by very low follow-up [31].

Meanwhile, a few studies conducted at mines and commercial farms were reporting much higher levels of sexual risk. For example, 29% of male migrant workers reported buying sex in the last year, compared with 4% in a household survey from 2006 [32,23]. In commercial farm settings (mainly Mashonaland), a third of men reported multiple partners [33]. At commercial farms near Harare, 60% of married men but only 4% of married women admitted to extra-marital relationships [34].

#### Explaining a declining epidemic

From roughly 1998 to 2003, multiple data sources pointed to a declining HIV epidemic. In antenatal clinic (ANC) trends, the largest declines among 15–24 years old women (from 12 to 4.8%) occurred before 2003 [35,36]. The second round of Manicaland community surveys in 2003 also revealed markedly lower HIV prevalence, particularly among young women (49% lower) and young men (23% lower). Behavioural data suggested reductions in casual partnerships among young people, delay in sexual debut and high rates of reported condom use since 1999 [7]. After adjusting for high mortality and other factors, part of the HIV prevalence decline was attributed to a few indicators of individual behaviour change [6,7,9,21,37,38].

On a population level, however, these data are less convincing. The proportions reporting reductions in casual sex (49% for men, 22% for women), for example, did not correlate well to HIV prevalence declines by sex, which were greater among women. Explanations may include social desirability bias, or indirect mechanisms whereby prevalence among women varies in response to changes in male risk behaviour. The changing size of male populations related to migration again confounds analysis of transmission dynamics during this period.

Delaying sexual debut and avoiding older partners appeared to decrease individual risk, although this was thought unlikely to influence HIV transmission at population level without wider ranging behavioural changes throughout sexual networks [39]. HIV incidence data from Manicaland identified only multiple partners, having an unwell partner and reporting another STI as proximate determinants [40,41].

Other research generated hypotheses to explain trends observed from household samples. Changes in sexual partnerships, for example, were studied over the five survey rounds in Manicaland between 1998 and 2011 [42]. Multiple partnerships and nonmarital concurrency were reported much more frequently by men (34.2 and 11.9%) than women (4.6 and 1.8%), and all indicators declined by 60–70% over survey rounds. The distribution of reported sex acts and condom use also varied by partnership type and marital status [43]. Neither study, however, reported on the fraction of partnerships, concurrency, sex acts or condom use that intersected with sex work networks or may have taken place outside the study areas. There is limited and inconsistent evidence that exposure to interventions may have influenced risk behaviours (see supplementary appendix).

#### The economy, migrant labour and sex work as factors in HIV declines

Socioeconomic factors were also examined in relation to HIV infection in Manicaland where the largest decreases in HIV prevalence were seen in the top third of the wealth index distribution for both men and women [44]. A prospective household census conducted from 1998 to 2011 confirmed an increase in extreme poverty and found that HIV prevalence fell in all socioeconomic sub-groups [45]. Growing poverty was seen in a qualitative study to reduce men's ability to afford multiple partners [46].

**Fig. 4:**
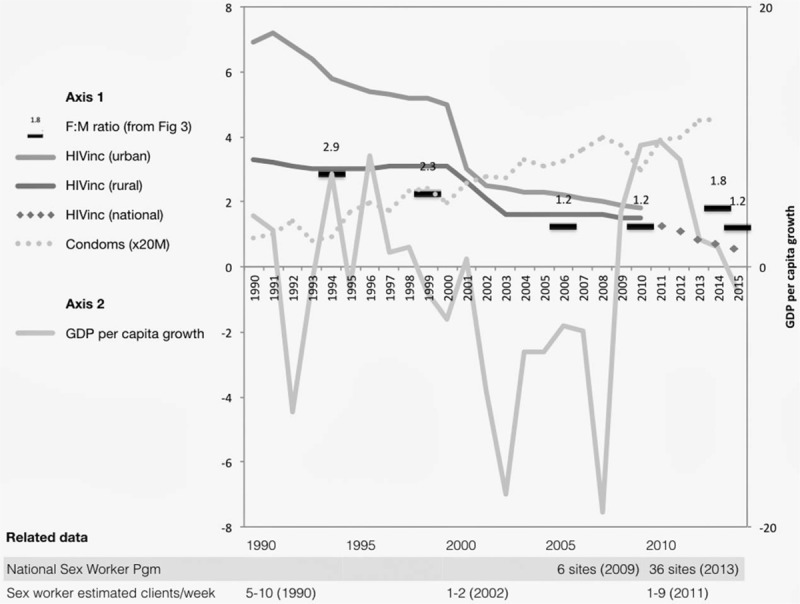
Estimated economic and HIV incidence trends, with condom distribution, sex worker programmatic and behavioural data.

The period of largest HIV prevalence declines (1998–2003) corresponded to the first half of a national economic crisis that continued to the hyperinflation of 2008. During this period, large and sustained shocks to the economy were having profound effects on migrant labour fluxes, which in turn likely reduced male demand for sex work (Fig. 4 and Table 1).

**Table 1:** Summary trends in economy, migration, sex work and HIV.

	1980–1998	1999–2008	2009–2015
Economy and migration	GDP growth averaged about 5.5% during 1980–1990. The general trend of the 1990s is that the economy showed signs of weakening, with cutbacks in production by manufacturing and other industries [5]. Migration began to slow (proxy measure: F : M ratio from 2.9 to 2.3).	The political and economic crises between 2000 and 2008 nearly halved GDP, raising poverty rates to more than 72% [5]. Migration declined and many more men were found at home during household surveys (F : M ratio reaches nadir at 1.2).	During 2009–2012, the economy rebounded, with growth rates averaging 8.7% per year. Growth slowed sharply during 2012–2015, because of shifts in trade and major droughts [5]. Migration variable (F : M ratio increases from 1.2 to 1.8 then slides back to 1.2).
Sex work demand, supply and client numbers	High levels of sex work were reported during the 1980s [1–3,31–33]. Early interventions with sex workers achieved partial coverage and raised condom use to moderately high levels [13,14]. Condom distribution continued to rise steadily across all the three periods.	Demand for sex work declined abruptly as unemployment rose, migrant workers returned home an incomes fell [47,49]. Supply increased as more poor women turned to sex work [48,49]. Low demand and higher supply led to a sharp decline in client number [47].	Sex workers reported a return to higher numbers of clients from 2009 as the economy improved [52,53,56]. More recently, sex workers report falling demand and lower prices (CeSHHAR reports).
HIV incidence/prevalence	HIV incidence is estimated to have peaked in the early 1990s and prevalence by mid-1990s [9]. The general pattern for the 1990s is progressive decline in urban areas and flat but lower incidence in rural areas reflecting slower rates of secondary transmission [9].	Modelled incidence suggests an abrupt decline at the beginning of this period, then levelling off, again with similar urban/rural pattern [9].	Modelled HIV prevalence (Spectrum) is estimated to have levelled off [8]. HIV incidence from 2015/2016 survey was 0.48.

Several studies conducted during this period support a trend towards lower volume sex work (see supplementary appendix). Among sex workers recruited for intervention trials in mining areas and commercial farms, 69% reported fewer than 17 lifetime sex partners, 73% claiming fewer than nine in the previous year [47]. Prevalences of curable, short-term STIs were also low – 5% for syphilis, 1.7% for chlamydia, 1.9% for gonorrhoea – suggesting unusually low levels of sexual transmission within sex work networks during that time. Prevalences of chronic viral STIs, which accumulate over longer durations, were much higher – 55.7% (HIV) and 80.8% (HSV-2) [47].

Rural poverty was also driving larger numbers of women and girls into sex work at a time when economic stagnation was suppressing demand [48]. As a result, cheaper and longer term ‘transactional’ arrangements were replacing higher volume sex work. There may have been more women involved in sex work but client numbers reported by sex workers were much lower. During the economic collapse, rural sex work in Manicaland also became more diffuse and less professional, with sex frequently being sold for commodities in lieu of cash [49].

Male risk behaviour was also studied at workplaces [34]. HIV prevalence was 27.3% among male workers surveyed at mines and commercial farms; 48.4% reported ever having had sexual contact with a sex worker, 29.3% in the past year. HIV was more common among men who reported sex worker contact [adjusted odds ratio (aOR) 1.4] and was also strongly associated with self-reported genital ulceration in the previous 6 months (aOR 3.1). Genital ulceration in turn correlated highly with sex worker contact. Such data, supported by modelling, corroborate ongoing individual risk despite changes in employment that reduced aggregate demand for sex work [50].

On the programme side, the initial focus on sex work during the early to mid-1990s was not sustained. The near absence of prevention was documented by a study in Harare where no sex workers were found to practice consistent condom use, 86% were HIV positive and 34% were found to have at least one STI [51].

### Recent trends and an evolving national response (circa 2009–2016)

Zimbabwe's National Strategic Plan 2011–2015 emphasized combination HIV prevention, bringing a number of general population services, including antiretroviral therapy (ART), prevention of mother-to-child transmission, voluntary medical male circumcision and continued condom programming, to scale (see supplementary appendix). Importantly, these general population services were complemented by a scaled-up prevention response in sex work.

‘Sisters with a Voice,’ Zimbabwe's National Sex Work Programme, began in 2009 with 3 fixed urban sites and 13 part-time mobile highway sites, with the Centre for Sexual Health and HIV AIDS Research (CeSHHAR) as implementing and research partner. In 2011, an RDS study conducted in Mutare, Hwange and Victoria Falls confirmed that sex work was indeed increasing as Zimbabwe's economy began to show signs of recovery [52]. Sex workers reported relatively high client numbers compared with 5–10 years earlier, and inconsistent condom use. HIV prevalence ranged from 50 to 70%, of whom only 25–35% were on ART. Competition for clients was high despite examples of sex worker solidarity [53].

Sisters with a Voice expanded further in 2013 to 6 fixed and 30 mobile sites covering main urban areas and transport corridors nationwide. Complementary bridge group interventions reached long-distance truckers and condom distribution expanded (see supplementary appendix). For the first time since the 1990s, the national prevention response was addressing both primary and secondary transmission networks (Fig. 2). Moreover, the range of services offered to sex workers, now including ART and PrEP, arguably strengthened the earlier platform of peer-based outreach, condom promotion and STI screening [54,55].

Other studies highlight changing conditions, risk and vulnerability in sex work (see supplementary appendix). A Manicaland study in 2010 reported a relatively high median price of 10 US dollars per sex work transaction, evidence of rebounding client demand [56]. Client requests for condom use significantly predicted protected sex (*P* < 0.01), but clients paid 43% more for unprotected sex.

## Discussion

Zimbabwe's HIV epidemic has been extensively documented, from its explosive eruption, with incidence peaking around 1990, to a period of rapid decline about a decade later. Yet, the methods used to describe the epidemic, underlying assumptions and data sources changed markedly between early and later periods, complicating analysis of trends and attribution. This changing perspective – from monitoring a few key epidemiological variables among high-risk groups to a more diffuse study of sexual behaviour across an entire population – also served to justify a radical reorientation of the epidemic response. Although the early focus was on slowing transmission where it was spreading the fastest, later emphasis on ‘universal access’ aimed to reach virtually everyone.

One of the challenges for understanding Zimbabwe's HIV epidemic is to tease out what was going on among sex workers and their clients during more than a decade of a largely generalized response. The few available articles on sex work during this period (1998–2008) suggest that sex workers had far fewer clients than reported in earlier (and later) studies. Despite high HIV prevalence reflecting years of exposure, other STIs among sex workers were at historically low levels for Zimbabwe, and much lower than in other countries. These findings suggest lower levels of sexual transmission at a time when the Zimbabwean economy was in a prolonged period of contraction (Fig. 4). In terms of HIV/STI transmission, these observations are potentially of great importance, given the central role of partner change in sexual transmission (see supplementary appendix).

Some of these changes can be perceived indirectly in data from household surveys. The proportions of men to women found at home during ZDHS rounds in 2006 and 2010 (∼80%) were far higher than in 1999 (less than 50%), reflecting a seismic shift in migration patterns affecting up to a third of the male population. Similarly, the most significant change in sexual behaviour reported by those men – a decrease in nonregular partners – may be related to less money, fewer opportunities and perhaps less unmet need, while at home. Although nearly a third of men reported buying sex in the last year when interviewed at a migrant workplace in 2001, only 4% did so when asked at home during the 2006 ZDHS. It is important to be aware of pitfalls in directly comparing these data from different surveys. Still, the orders of magnitude are striking and argue for better understanding of such extreme heterogeneity in conditions that are known to drive epidemics.

This interpretation of historical epidemiological evidence also supports the continued centrality of sex work and population mobility as epidemic drivers. When HIV prevention in sex work is neglected, even in advanced epidemics such as Zimbabwe's, high incidence generated in migrant destination areas can serve as an efficient transmission pump to sustain prevalence among the wider population. When the pump jams, as a result of economic shocks or other structural factors, the force of new infections subsides and overall prevalence levels decline. Yet, structural conditions are often cyclical, and epidemics can move in either direction.

What remains critical is sustaining interventions to counter key epidemic drivers (see supplementary appendix). Simply raising consistent condom use in sex work, while scaling up ART for all, has been estimated to reduce HIV prevalence up to 46% more than ART scale-up alone. Recent Zimbabwe data suggest that a more comprehensive package of HIV/STI services for sex workers would likely have even greater impact on HIV transmission [54,55]. It is plausible that the recent scale-up of Zimbabwe's National Sex Worker Programme, together with broader Combination HIV Prevention, are simultaneously slowing primary transmission ‘upstream’ in sex work networks and secondary transmission ‘downstream’ among the general population. The combined impact of such complimentary efforts may well explain recently reported low HIV incidence.

Social and economic factors, including migration and population mobility, continue to fluctuate, within and across countries in an increasingly globalized world. These structural factors in turn influence critical drivers of sexually transmitted epidemics, particularly in sex work networks and related secondary transmission. Understanding these changes, and monitoring the intervention response, require epidemiologic methods that can disentangle different network streams from aggregate national data, identify key transmission drivers and provide reliable estimates and projections for prevention planning. In today's context of flat HIV funding, such sharper epidemiologic approaches are more important than ever.

## Acknowledgements

### Conflicts of interest

There are no conflicts of interest.

## Supplementary Material

Supplemental Digital Content
