# Supplementary material for: Economy, migrant labour and sex work: interplay of HIV epidemic drivers in Zimbabwe over three decades
Source: AIDS. 2018 Dec 3;33(1):123–31. doi: 10.1097/QAD.0000000000002066 (PMC6415983; doi:10.1097/QAD.0000000000002066)
Supplement: Supplemental Digital Content [file aids-33-123-s001.doc]

**Supplementary appendix**

The following additional references support sections of the text.

**Introduction**

The importance of sex work and population mobility in early, concentrated HIV epidemics, and for other sexually transmitted infections (STIs), is widely recognised. [1-6] Predominantly male migration (high male-to-female ratio), thought to increase demand for sex work, was associated with larger HIV epidemics. [1] With high rates of partner change and secondary transmission via ‘bridge’ populations, HIV transmission potential in sex work was estimated to be several hundred times higher than in lower-risk transmission networks. [7,8] More recent evidence and modelling suggest that such large transmission differentials persist even in advanced generalised epidemics. [9-14]

**Results**

Phylogenetic and historical evidence suggest that HIV moved with migrant labour from Léopoldville (Kinshasa) to the Copper Belt of Eastern Congo during the half-century before it first appeared in Zimbabwe. [15] By the time AIDS was recognised, conditions for efficient spread through migrant networks, amplified by long-distance trucking, were well established across the region. [16,17]

Male condom distribution, free and socially marketed, increased almost 3-fold between 1990 and 1999 to 60 million (see supplementary appendix). [18,19]

As HIV became entrenched in diverse communities across the country, the attention of both researchers and programmes shifted to the general population. In 2000 WHO defined three epidemic states for surveillance purposes – low, concentrated and generalised – with recommendations for routine data collection. [20] These surveillance classifications, following reported HIV declines in Uganda, influenced programming and policy recommendations across generalised epidemics of Eastern and Southern Africa. [21-25]

There is limited and inconsistent evidence that exposure to interventions may have influenced risk behaviours. [26-28] Voluntary counselling and testing (VCT) was associated with fewer new partners reported by women, especially those testing positive. No behavioural risk reduction was seen among men following VCT, however, including no increase in consistent condom use with regular or non-regular partners. [29]

Several studies conducted during this period support a trend towards lower-volume sex work. [30-34]

Zimbabwe’s National Strategic Plan 2011-2015 emphasised Combination HIV prevention, bringing a number of general population services, including ART, PMTCT, VMMC, and continued condom programming, to scale. [35]

Complementary bridge group interventions reached long-distance truckers and condom distribution expanded. [35,18,19]

Several recent studies shed light on changing conditions, risk and vulnerability factors and their interactions in the context of sex work. [36-41]

**Discussion**

While the early focus was on slowing transmission where it was spreading fastest, later emphasis on ‘universal access’ aimed to reach virtually everyone. [42]

These findings suggest lower levels of sexual transmission at a time when the Zimbabwean economy was in a prolonged period of contraction. In terms of HIV/STI transmission, these observations are potentially of great importance given the central role of partner change in sexual transmission. [9-14]

What remains critical is sustaining interventions to counter key epidemic drivers. [9-14] Simply raising consistent condom use in sex work, while scaling up ART for all, has been estimated to reduce HIV prevalence up to 46% more than ART scale-up alone. [10] Recent Zimbabwe data suggest that a more comprehensive package of HIV/STI services for sex workers would likely have even greater impact on HIV transmission. [43,44] It is plausible that the recent scale-up of Zimbabwe’s National Sex Worker Programme, together with broader Combination HIV Prevention, are simultaneously slowing primary transmission ‘upstream’ in sex work networks and secondary transmission ‘downstream’ among the general population. The combined impact of such complimentary efforts may well explain recently reported low HIV incidence. [45]

**References**

1. Over, M. The effects of societal variables on urban rates of HIV infection in developing countries: An exploratory analysis. in: Confronting AIDS: Evidence from the Developing World. ed. Ainsworth M, Franzen L, Over M, 40-51. Brussels, European Commission, 1998.
2. Brandt AM. No magic bullet: a social history of venereal disease in the United States since 1880. New York, Oxford University Press, 1987.
3. Brunham RC, Plummer FA. A general model of sexually transmitted disease epidemiology and its implications for control. Med Clin North Am 1990; 74:1339–1352.
4. Cameron DW, Simonsen JN, D’Costa LJ, et al. Female to male transmission of human immunodeficiency virus type 1: risk factors for seroconversion in men. Lancet 1989; ii 403-407.
5. Jessamine PG et al. Human immunodeficiency virus, genital ulcers and the male foreskin: synergism in HIV-1 transmission. Scandinavian Journal of Infectious Diseases, 1990;69: S181– S186.
6. Voeten HA, Vissers DC, Gregson S, Zaba B, White RG, de Vlas SJ, Habbema JD. Strong association between in-migration and HIV prevalence in urban sub-Saharan Africa. Sex Transm Dis. 2010;37(4):240-3. doi: 10.1097/OLQ.0b013e3181c3f2d0.
7. Moses S, Plummer FA, Ngugi EN, Nagelkerke NJD, Anzala AO, Ndinya-Achola JO. Controlling HIV in Africa: effectiveness and cost of an intervention in a high-frequency STD transmitter core group. AIDS 1991;5:407-11.
8. World Bank. Confronting AIDS: Public Priorities in a Global Epidemic. New York: Oxford University Press, 1997.
9. Chen L, Jha P, Stirling B, Sgaier SK, Daid T, et al. Sexual Risk Factors for HIV Infection in Early and Advanced HIV Epidemics in Sub-Saharan Africa: Systematic Overview of 68 Epidemiological Studies. PLoS ONE 2007; 2(10): e1001. doi:10.1371/journal.pone.0001001
10. Steen R, Hontelez JA, Veraart A, White RG, de Vlas SJ. Looking upstream to prevent HIV transmission: can interventions with sex workers alter the course of HIV epidemics in Africa as they did in Asia? AIDS 2014;28: 891–899. doi: 10.1097/QAD.0000000000000176 PMID: 24401648
11. Mishra S, Sgaier SK, Thompson LH, Moses S, Ramesh BM, et al. HIV Epidemic Appraisals for Assisting in the Design of Effective Prevention Programmes: Shifting the Paradigm Back to Basics. PLoS ONE. 2012;7(3): e32324. doi:10.1371/journal.pone.0032324
12. Mishra S, Steen R, Gerbase A, Lo Y-R, Boily M-C. Impact of High-Risk Sex and Focused Interventions in Heterosexual HIV Epidemics: A Systematic Review of Mathematical Models. PLoS ONE. 2012;7(11): e50691. doi:10.1371/journal.pone.0050691
13. Mishra S, Pickles M, Blanchard JF, Moses S, Shubber Z, Boily M-C, et al. Validation of the modes of transmission model as a tool to prioritize HIV prevention targets: a comparative modelling analysis. PLoS One 2014; 9: e101690. doi: 10.1371/journal.pone.0101690 PMID: 25014543
14. Steen R, Wheeler T, Gorgens M, Mziray E, Dallabetta G. Feasible, Efficient and Necessary, without Exception – Working with Sex Workers Interrupts HIV/STI Transmission and Brings Treatment to Many in Need. PLoS ONE 2015; 10(10): e0121145. doi:10.1371/journal.pone.0121145
15. Faria NR, Rambaut A, Suchard MA et al. The early spread and epidemic ignition of HIV-1 in human populations. Science. 2014; 346(6205): 56–61. doi:10.1126/science.1256739.
16. Conover T. Trucking through the AIDS belt. The New Yorker, August 16, 1993.
17. Abdool Karim Q, Abdool Karim S. The evolving HIV epidemic in South Africa. Int J Epidemiol 2002;31(1):37-40. DOI: https://doi.org/10.1093/ije/31.1.37
18. Zimbabwe AIDS response country progress reports and National AIDS Council Annual Reports. www.nac.org.zw/ documents/documents-and-reports. Accessed 26 July 2017.
19. DKT. <http://www.dktinternational.org/publications-resources/contraceptive-social-marketing-statistics/>. Accessed 26 July 2017.
20. UNAIDS/WHO. Guidelines for Second Generation HIV Surveillance. WHO/CDS/CSR/EDC/2000.5 <http://www.who.int/hiv/pub/surveillance/en/cds_edc_2000_5.pdf>]
21. Stoneburner RL, Low-Beer D. Population-level HIV declines and behavioral risk avoidance in Uganda. Science 304: 714–718.
22. Gray RH, Wawer JM (2008) Reassessing the hypothesis on STI control for HIV prevention. Lancet. 2004;371:2064–2065.
23. Shelton J (2008) Counselling and testing for HIV prevention. Lancet 372: 273–275.
24. Hallett TB, Aberle-Grasse J, Bello G, Boulos LM, Cayemittes MP, Cheluget B, et al. Declines in HIV prevalence can be associated with changing sexual behavior in Uganda, urban Kenya, Zimbabwe, and urban Haiti. Sex Transm Infect. 2006;82 Suppl 1:i1-8.
25. Halperin DT, Mugurungi O, Hallett TB, Muchini B, Campbell B, et al. A Surprising Prevention Success: Why Did the HIV Epidemic Decline in Zimbabwe? PLoS Med. 2011;8(2): e1000414. doi:10.1371/ journal.pmed.1000414
26. Gregson S, Nyamukapa C, Schumacher C, Mugurungi O, Benedikt C, Mushati P, et al. Did national HIV prevention programs contribute to HIV decline in Eastern Zimbabwe? Evidence from a prospective community survey. Sex Transm Dis. 2011;38:475-82.
27. Gregson S, Mushati P, Grusin H, Nhamo M, Schumacher C, Skovdal M, et al. Social capital and women's reduced vulnerability to HIV infection in rural Zimbabwe. Popul Dev Rev. 2011;37:333-59.
28. Hallett TB, Dube S, Cremin I, Lopman B, Mahomva A, Ncube G, et al. The role of testing and counselling for HIV prevention and care in the era of scaling-up antiretroviral therapy. Epidemics. 2009;1:77-82.
29. Cremin I, Nyamukapa C, Sherr L, Hallett TB, Chawira G, Cauchemez S, et al. Patterns of self-reported behavior change associated with receiving voluntary counselling and testing in a longitudinal study from Manicaland, Zimbabwe. AIDS Behav. 2010;14:708-15.
30. Cowan FM, Hargrove JW, Langhaug LF, Jaffar S, Mhuriyengwe L, Swarthout TD, et al. The appropriateness of core group interventions using presumptive periodic treatment among rural Zimbabwean women who exchange sex for gifts or money. J Acquir Immune Defic Syndr. 2005;38:202-7.
31. Cowan FM, Pascoe SJ, Barlow KL, Langhaug LF, Jaffar S, Hargrove JW, et al. Association of genital shedding of herpes simplex virus type 2 and HIV-1 among sex workers in rural Zimbabwe. J. AIDS 2006;20(2):261-7.
32. Cowan FM, Pascoe SJ, Barlow KL, Langhaug LF, Jaffar S, Hargrove JW, et al. A randomized placebo-controlled trial to explore the effect of suppressive therapy with acyclovir on genital shedding of HIV-1 and herpes simplex virus type 2 among Zimbabwean sex workers. Sex Transm Infect. 2008;84:548-53.
33. Save the Children. The livelihoods of commercial sex workers in Binga. Report Date: March 27, 2002
34. Elmes J, Nhongo K, Skovdal M, et al. PCS1.03: The changing nature and contribution of sex work to HIV transmission during the economic crisis in Zimbabwe [oral presentation]. In: Pre-congress symposium, ISSTDR STI & AIDS World Congress, Vienna, Austria, 2013
35. Zimbabwe National HIV and AIDS Strategic Plan 2006-2010. 2006 Harare.
36. Busza J., Mtetwa S., Chirawu P. and Cowan F. Triple jeopardy: adolescent experiences of sex work and migration in Zimbabwe. Health Place 2014;28:85-91.
37. Scorgie F, Nakato D, Harper E, Richter M, Maseko S, Nare P, et al. 'We are despised in the hospitals': sex workers' experiences of accessing health care in four African countries. Cult Health Sex. 2013;15:450-65.
38. Elmes J, Nhongo K, Ward H, Hallett T, Nyamukapa C, White PJ, Gregson S et al. The Price of Sex: Condom Use and the Determinants of the Price of Sex Among Female Sex Workers in Eastern Zimbabwe. JID 2014;210(S2):569–78
39. Masvawure T. 'I just need to be flashy on campus': female students and transactional sex at a university in Zimbabwe. Cult Health Sex 2010;12(8):857-70.
40. Masvawure T. The role of 'pimping' in the mediation of transactional sex at a university campus in Zimbabwe. Afr J AIDS Res. 2011;10:165-71.
41. Nkomazana N, Maharaj P. The prevalence of condom use among university students in Zimbabwe: implications for planning and policy. J Biosoc Sci. 2013;45:643-59.
42. Buzdugan R, Benedikt C, Langhaug L, et al Population-Level Impact of Zimbabwe’s National Behavioural Change Programme. J Acquir Immune Defic Syndr 2014;67:e134–e141
43. Hargreaves JR, Mtetwa S, Davey C, Dirawo J, Chidiya S, Benedikt C, et al. Cohort analysis of programme data to estimate HIV incidence and uptake of HIV-related services among female sex workers in Zimbabwe, 2009-14. J Acquir Immune Defic Syndr. 2016;72(1):e1–e8.
44. Cowan FM, Davey C, Fearon E, et al. The HIV care cascade among female sex workers in Zimbabwe: results of a population-based survey from the Sisters Antiretroviral therapy Programme for Prevention of HIV, an Integrated Response (SAPPH-IRe) Trial. J Acquir Immune Defic Syndr 2017;74:375–382
45. Zimbabwe population-based HIV impact assessment ZIMPHIA 2015–2016. http://phia.icap.columbia.edu/countries/zimbabwe/. accessed 15 April 2018.
